# Overexpression of UDP-Glucose 4-Epimerase Is Associated with Differentiation Grade of Gastric Cancer

**DOI:** 10.1155/2019/6325326

**Published:** 2019-11-20

**Authors:** Maria de Fátima Deodato de Souza, Antônio Felix da Silva Filho, Amanda Pinheiro de Barros Albuquerque, Michael Williams Leal Quirino, Mário Sérgio de Souza Albuquerque, Marina Ferraz Cordeiro, Mário Rino Martins, Ivan da Rocha Pitta, Antônio Roberto Lucena-Araujo, Maira Galdino da Rocha Pitta, Moacyr Jesus Barreto de Melo Rêgo

**Affiliations:** ^1^Laboratório de Imunomodulação e Novas Abordagens Terapêuticas-LINAT/Núcleo de Pesquisa em Inovação Terapêutica Suely Galdino-NUPIT SG, Universidade Federal de Pernambuco, Recife, PE, Brazil; ^2^Universidade Federal do Vale do São Francisco, Colegiado de Medicina, Paulo Afonso, BA, Brazil; ^3^Hospital do Câncer de Pernambuco-HCP, Recife, PE, Brazil; ^4^Departamento de Genetica, Universidade Federal de Pernambuco, Recife, PE, Brazil

## Abstract

The UDP-glucose 4-epimerase (GALE) is a glycosyltransferase, which acts on protein and lipid glycosylation in normal and neoplastic cells. This study is aimed at investigating the differential tissue expression of GALE and its possible association with clinical-pathological parameters and the outcome of gastric adenocarcinoma patients. Seventy-one patients were evaluated in relation to GALE expression by immunohistochemistry. Our results showed that 48 (67.6%) patients were GALE positive and 23 (32.4%) negative. Positive staining was present on well-differentiated and moderate-differentiated histological grade of gastric adenocarcinomas (*p* < 0.0001). There was no significant association with outcome parameters (*p* > 0.05). Besides that, our results corroborated with the validation cohort analysis, where the expression of GALE mRNA was also associated with the histological grade (*p* < 0.001). These results suggest a possible use of this enzyme as a biomarker for well and moderately differentiated tumors.

## 1. Introduction

Gastric cancer (GC) has low survival rates and represents the second leading cause of death associated with cancer worldwide. This neoplasia presents heterogeneous characteristics and different biological behaviors, besides variations in therapeutic response and clinical course [1–4]. Discovery of new biomarkers for diagnosis and a segment of GC can optimize its therapeutic strategies [5].

Glycosylation is a common post-translational and/or co-translational biochemical event in protein and lipid synthesis. It is an important cellular mechanism often altered in several types of cancer. The first step of glycosylation reactions is determined by glycosyltransferases (GTs) [6]. Changes in GTs expression leads to an altered glycosylation pattern, contributing to cancer progression. Therefore, GTs can represent potential biomarker candidates [7].

One of these GTs, UDP-glucose 4-epimerase (also called UDP-galactose 4-epimerase), is a member of the short chain dehydrogenase/reductase superfamily [8]. Functionally, UDP-glucose 4-epimerase catalyzes the reversible conversion between UDP-galactose (UDP-Gal) and UDP-glucose (UDP-Glc) in the third step of the Leloir pathway in galactose metabolism. Additionally, this GT promotes the interconvention of UDP-N-acetylgalactosamine (UDP-GalNAc) in UDP-N-acetylglucosamine (UDP-GlcNAc) [9–11]. The expression of UDP-glucose 4-epimerase was found in hepatomas, lymphoma, and thyroid cancer. The products of its metabolic activity may perform a significant role in cell recognition, signaling, progression, and metastasis of cancer cells [12–14].

Thus, this study is aimed at analyzing GALE staining on tissue samples of GC to investigate whether its expression is associated with pathological clinical parameters and with overall survival rates and disease-free survival.

## 2. Materials and Methods

### 2.1. Samples

Seventy-one patients with primary gastric adenocarcinoma, diagnosed between 2013 and 2016, were selected from the Service Registry of the Pernambuco Cancer Hospital (HCP). The clinical specimens were previously fixed in buffered formalin and embedded in paraffin. The following variables were collected in medical charts: age, sex, tumor type, location, pathological staging, Lauren classification, treatment type, lymph node number and location, positive lymph nodes, recurrence, metastasis, and death. The Human Ethics Committee of HCP approved this work (CAAE: 39976214.90000.5205).

### 2.2. Immunohistochemistry

Following the methods of Albuquerque et al. (2018) [15], biopsy slices were deparaffinized with xylol and rehydrated in graded ethanol. Antigen retrieval was done using citrate buffer in a microwave. Endogenous peroxidase block was performed with hydrogen peroxide 3%, followed by blocking the nonspecific binding (phosphate-buffered saline 1%) bovine serum albumin (PBS-BSA). Samples were incubated with polyclonal primary antibody anti-UDP-glucose 4-epimerase (CUSABIO, 1 : 100) overnight at 4°C. The amplification system (Easylink On, ImmPRESS™, and DAKO EnVision™) was applied, and the reaction was visualized with diaminobenzidine (DAB-H_2_O_2_). The positive control used was breast cancer tissue according to the antibody manufacturer's designation (CUSABIO TECHNOLOGY LLC), and negative controls were established by replacing the primary antibody with anti-human IgG antibody (DAKO) (supplementary file ([Supplementary-material supplementary-material-1])).

### 2.3. Image Analysis

Histomorphological analysis was performed with an integrated image system (BIOPTICA B20) microscope coupled to a CMOS camera (2584 × 1936 pixel resolution) with ISCapture image capture software. Samples that showed enzyme marking in more than 10% of the neoplastic cells in different degrees of intensity were considered positive and those that did not present were negative. The enzyme cell location, cytoplasmic, membrane, and perinuclear and nuclear combinations were associated with clinical-pathological parameters and outcome parameters.

### 2.4. Analysis of the Validation Cohort

The validation cohort analyzed in this study was extracted from the cBioPortal PC genomic (http://www.cbioportal.org) (Cerami et al., 2012; Gao et al., 2013). The Stomach Adenocarcinoma (TCGA, Provisional), comprising 411 patients, was used for analysis of the GALE expression. Briefly, the GALE mRNA expression value was compared with clinical-pathological data (age, gender, lymph node involvement, histological grade, Lauren classification, nodal status, H. pylori infection, surgical staging, radiotherapy, and relapse) and with outcome parameters (overall survival and disease-free survival), and statistical association was analyzed using Fisher's exact test and Kaplan-Meyer curves with a long-rank test. Statistical analysis was performed using GraphPad Prism (version 7) software.

### 2.5. Statistical Analysis

Fisher's exact test was performed in GraphPad Prism version 6.0. *p* < 0.05 and was considered significant. Analysis of outcome was evaluated through Kaplan-Meyer curves with a long-rank test. Multivariate logistic regression analysis was performed using STATA.

## 3. Results

**Figure 1:**
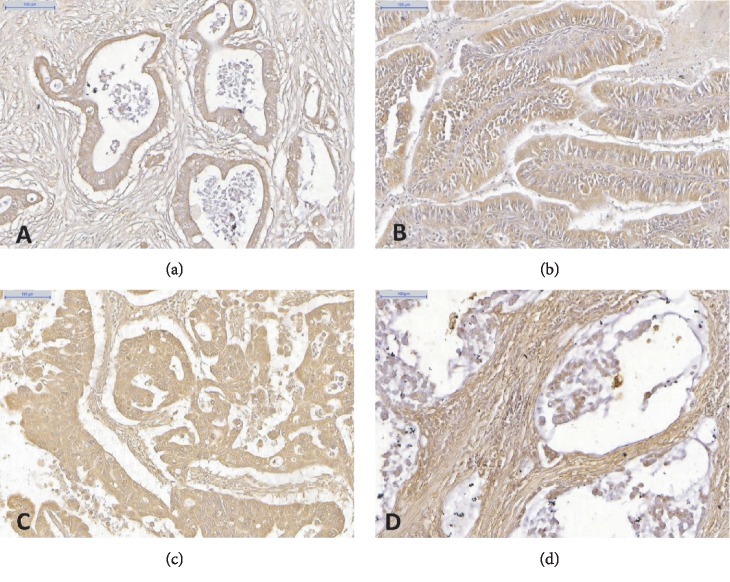
Histological types of gastric adenocarcinoma evidenced by GALE immunohistochemistry. Tubular region (a), papillary region (b), poorly differentiated region (c), and delimited mucin region (d). Scale bar: 100 *μ*m.

GC patients included in this study had a mean age of 59.4 ± 12.9 (range = 30‐89) years; 47 (66.1%) were male and 24 (33.9%) were female. Immunohistochemical analysis revealed that of the 47 male cases, 34 (70.83%) were GALE positive and 13 (29.17%) were GALE negative. Of the 24 female cases, 14 (58.33%) were GALE positive and 10 (41.67%) were GALE negative. In total, 48 (67.6%) samples were positive and 23 (32.4%) were negative for GALE. GC histological types positive to GALE corresponded to tubular (Figure 1(a)), papillary (Figure 1(b)), poorly differentiated (Figure 1(c)), and mucinous (Figure 1(d)).

**Figure 2:**
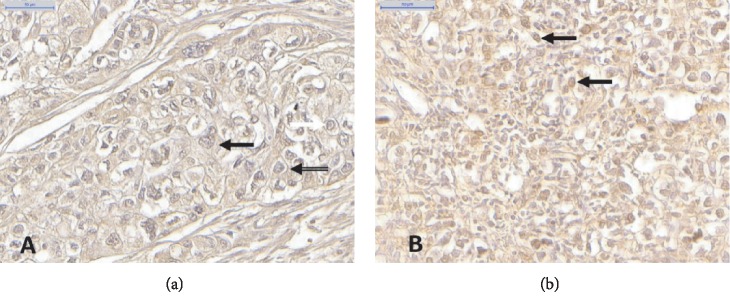
Immunohistochemistry of GC samples. Cytoplasmic staining (black arrow), membrane staining (hollow arrow), perinuclear staining (white arrow) (a), and nuclear staining (black arrows) (b). Scale bar: 50 *μ*m.

All samples that presented tubular lesions were GALE positive with a predominance of cytoplasmic staining, whereas most poorly differentiated lesions were negative. GALE cellular staining was found to be cytoplasmic in 24 samples (33.8%), nuclear in 3 (4.2%), and combinations of cytoplasmic, nuclear, perinuclear, and membrane staining in 44 (61.9%) (Figure 2).

In addition, 35 samples (49.3%) of the 71 patients investigated presented an adjacent normal tissue, in which 29 (82.9%) were GALE positive and 6 (17.1%) were negative (supplementary file). Here, GALE staining was observed in the basal layer of the ducts and in production cells of the gastric glands. This staining was found predominantly in the cytoplasm and membrane of epithelial cells.

GALE staining was also investigated in relation to its association with GC clinical-pathological parameters. There was a significant association with well-differentiated histologic classification (*p* < 0.0001) when compared to poorly differentiated tissue; there was also a significant association of intestinal type (*p* = 0.0328) when compared to diffuse type (Lauren classification) (Table 1). Additional analysis regarding age, sex, type of surgery, initial treatment, surgical staging, lymph node involvement, nodal status, chemotherapy, radiotherapy, H. pylori infection, and relapse was not significant. Associations between cellular location staining and clinical-pathological parameters were not statistically significant either (*p* > 0.05).

It was also observed that GALE was able to predict Lauren's classification, exercising a protective effect in relation to the diffuse type (Table 2). This effect was independently maintained in a multivariate analysis model, considering age and surgical staging (Table 2).

Associations with overall survival were 334 days for the positive group and 514 days for the negative group (*p* = 0.665). In relation to disease-free survival, the positive and negative groups had a mean survival of 11 and 19 months, respectively (*p* = 0.228), and were not statistically significant (supplementary file).

We analyzed a validation cohort composed of 411 patients with gastric adenocarcinoma extracted from the cBioPortal PC genome. GALE mRNA expression was associated with histological grade (*p* < 0.001). Additional analysis concerning clinical and pathological parameters (age, sex, lymph node involvement, nodal state, H. pylori infection, surgical staging, radiotherapy, and relapse) and outcome parameters (overall survival and disease-free survival) was not statistically significant (*p* > 0.05), ratifying our data (supplementary file).

## 4. Discussion

This study demonstrated that the GALE expression is associated with gastric adenocarcinomas with well and moderately differentiated histological grades. GALE staining was predominantly cytoplasmic in tubular lesions, and there was also membrane, perinuclear, and nuclear staining to that and other histological patterns. These include papillary, mucinous, and poorly differentiated. Moreover, analysis of normal segments revealed GALE positivity in the gastric glands with cytoplasmic and membrane staining profiles.

UDP-galactose is an intermediate in the metabolism of D-galactose (the Leloir pathway, a known amphibolic metabolic pathway), which is conserved from Escherichia coli in humans. Alterations in GALE activity can lead to an accumulation of UDP-galactose, causing stress, stopping cell growth, and stopping cell lysis in a complex medium [16, 17].

Lee and collaborators proposed the mechanism by which GALE absence causes cellular growth to stop. This author made use of GALE negative strains of Escherichia coli and notes that bacteriostasis in the presence of D-galactose directly comes from pyrimidine deficiency or an unbalanced nucleotide pool. This might be because enough uridine and cytidine are imported from the media to support RNA synthesis, yet do not suffice in making enough UDP intermediates, such as UDP-glucose for peptidoglycan synthesis [17].

In our study, GALE expression in well and moderately differentiated gastric adenocarcinomas can be related to a less aggressive cellular profile, corroborating the association with the intestinal type of Lauren classification. However, Mitteldorf et al. observed that GALE mRNA expression was significantly different in benign and malignant samples of the thyroid, being overexpressed in papillary thyroid carcinoma [13]. It is worth mentioning that these studies compared distinct tumors and targets at different molecular levels. However, papillary thyroid carcinoma is a well-differentiated tumor, and that is in agreement with our results.

Additionally, we found positive samples in adjacent normal tissue areas. According to their normal activity, the enzyme is a component of these cells acting on the metabolism of galactose and influencing the glycoprotein and glycolipid biosynthesis [11]. Associations with clinical and pathological parameters and outcome in relation to GALE mRNA expression from the validation cohort corroborated our data regarding GALE protein expression in gastric adenocarcinoma tissues.

## 5. Conclusion

This study is pioneering for evaluating the GC staining of GALE, showing an association of glycosyltransferase with well-differentiated tumors. In addition, further studies are needed to complement these initial findings in order to establish GALE as a biomarker of good prognosis to GC.

## Figures and Tables

**Table 1:** Association between UDP glucose 4-epimerase staining with the main clinical-pathological characteristics classified by immunohistochemistry in 71 samples of primary tumor of gastric cancer.

Clinical and pathological parameters	UDP-glucose 4-epimerase (+)	UDP-glucose 4-epimerase (-)	*p* value
	*n* (%)	*n* (%)	
Age (years)^a^			0.453
<60	22 (30.9)	13 (18.3)	
≥60	26 (36.6)	10 (14.1)	
Gender^b^			0.287
Female	14 (19.7)	10 (14.1)	
Male	34 (47.9)	13 (18.3)	
Initial treatment^c^			0.166
I	43 (60.6)	23 (32.3)	
III	5 (7.0)	0 (0)	
Surgical staging^d^ (TNM)			0.151
(I and II)	10 (14.1)	9 (12.7)	
(III and IV)	38 (53.5)	14 (19.7)	
Lymph node involvement^e^			0.299
Yes	32 (45.0)	12 (16.9)	
No	16 (22.5)	11 (15.5)	
Nodal status^f^			0.599
>0.3	19 (26.8)	7 (9.8)	
<0.3	29 (40.8)	16 (22.5)	
Histological grade^g^			<0.0001
GI+GII	32 (45.0)	3 (4.2)	
GIII	16 (22.5)	20 (28.2)	
Lauren classification^h^			0.0328
Intestinal	26 (38.2)	7 (10.2)	
Diffuse	19 (28)	16 (23.5)	
Chemotherapy^i^			0.452
Yes	28 (39.4)	11 (15.5)	
No	20 (28.2)	12 (16.9)	
Radiotherapy^j^			1.000
Yes	15 (21.1)	7 (9.8)	
No	33 (46.5)	16 (22.5)	
Relapse^l^			0.220
Yes	8 (11.3)	7 (9.8)	
No	40 (56.3)	16 (22.5)	
H. pylori infection^m^			0,707
Yes	7 (10.4)	2 (3.0)	
No	37 (55.2)	21 (31.3)	

Fisher's exact test. ^h^Lauren Classification analyzed in 68 cases. ^m^H. pylori infection was analyzed in 67 cases.

**Table 2:** Univariate and multivariate logistic regression analysis of GALE staining.

	Univariate				Multivariate			
Variable	OR	95% CI		*p* value	OR	95% CI		*p* value
Age (y): <60 versus ≥60	0.8	0.31	2.04	0.642	0.83	0.31	2.24	0.718
Surgical staging	0.7	0.24	2.02	0.51	0.83	0.26	2.56	0.747
GALE	0.32	0.11	0.92	0.036	0.33	0.11	0.98	0.04

## Data Availability

The data, in free formats, used to support the findings of this study are available from the corresponding author upon request.

## Supplementary Materials

Supplementary Materials Supplementary Figure 1: UDP-glucose 4-epimerase staining at the breast cancer tissue. Negative (A) and positive control (B). Scale bar: 50 *μ*m. Supplementary Figure 2: GALE immunohistochemistry. A) Positive adjacent normal gastric tissue (A), negative adjacent normal gastric tissue (B). Scale bar: 50 *μ*m. Supplementary Table 1: paired comparison of GALE staining in neoplastic cells and gastric nontransformed adjacent tissue. Supplementary Table 2: validation cohort. Association between the expression value of UDP-glucose 4-epimerase mRNA with the main clinical-pathological characteristics of patients with gastric adenocarcinoma. Supplementary Graph 1: associations with the outcome parameters. Overall survival (*p* = 0.665) (A) and disease-free survival (*p* = 0.228) (B) of the shunt cohort relative to the overall survival validation cohort (*p* = 0.605) (C) and disease-free survival (*p* = 0.637) (D).
