# Supplementary material for: Overexpression of UDP-Glucose 4-Epimerase Is Associated with Differentiation Grade of Gastric Cancer
Source: Dis Markers. 2019 Nov 20;2019:6325326. doi: 10.1155/2019/6325326 (PMC6886318; doi:10.1155/2019/6325326)
Supplement: Supplementary Materials — Supplementary Figure 1: UDP-glucose 4-epimerase staining at the breast cancer tissue. Negative (A) and positive control (B). Scale bar: 50 μm. Supplementary Figure 2: GALE immunohistochemistry. A) Positive adjacent normal gastric tissue (A), negative adjacent normal gastric tissue (B). Scale bar: 50 μm. Supplementary Table 1: paired comparison of GALE staining in neoplastic cells and gastric nontransformed adjacent tissue. Supplementary Table 2: validation cohort. Association between the expression value of UDP-glucose 4-epimerase mRNA with the main clinical-pathological characteristics of patients with gastric adenocarcinoma. Supplementary Graph 1: associations with the outcome parameters. Overall survival (p = 0.665) (A) and disease-free survival (p = 0.228) (B) of the shunt cohort relative to the overall survival validation cohort (p = 0.605) (C) and disease-free survival (p = 0.637) (D). [file 6325326.f1.docx]

**Supplementary Materials**


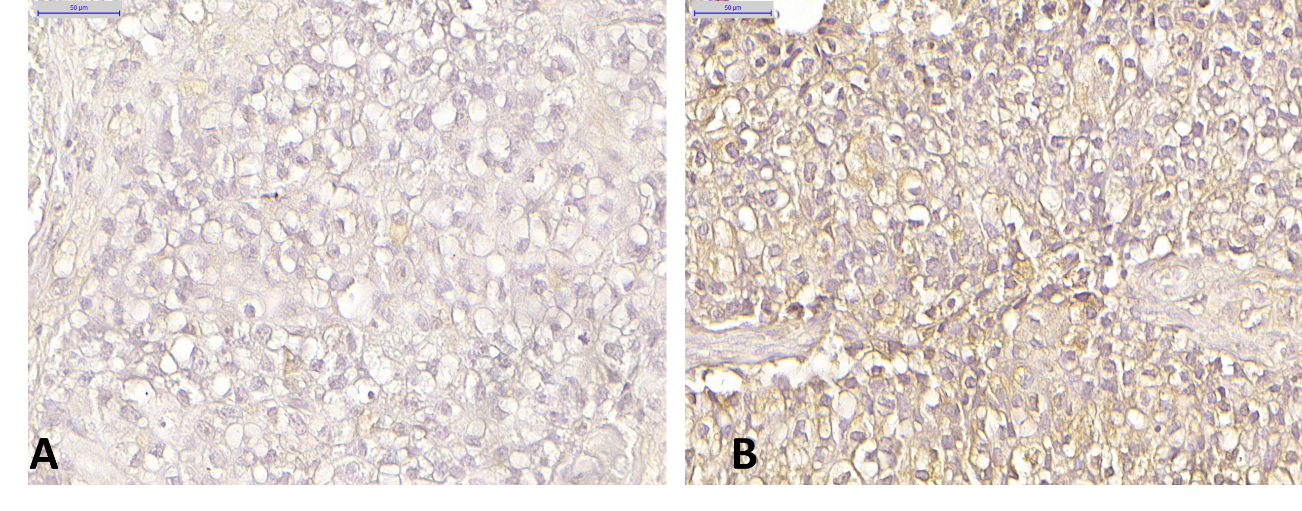


Supplementary Figure 1.UDP glucose 4-epimerase staining at the breast cancer tissue. Negative (A) and positive control (B). Scale bar: 50 μm.


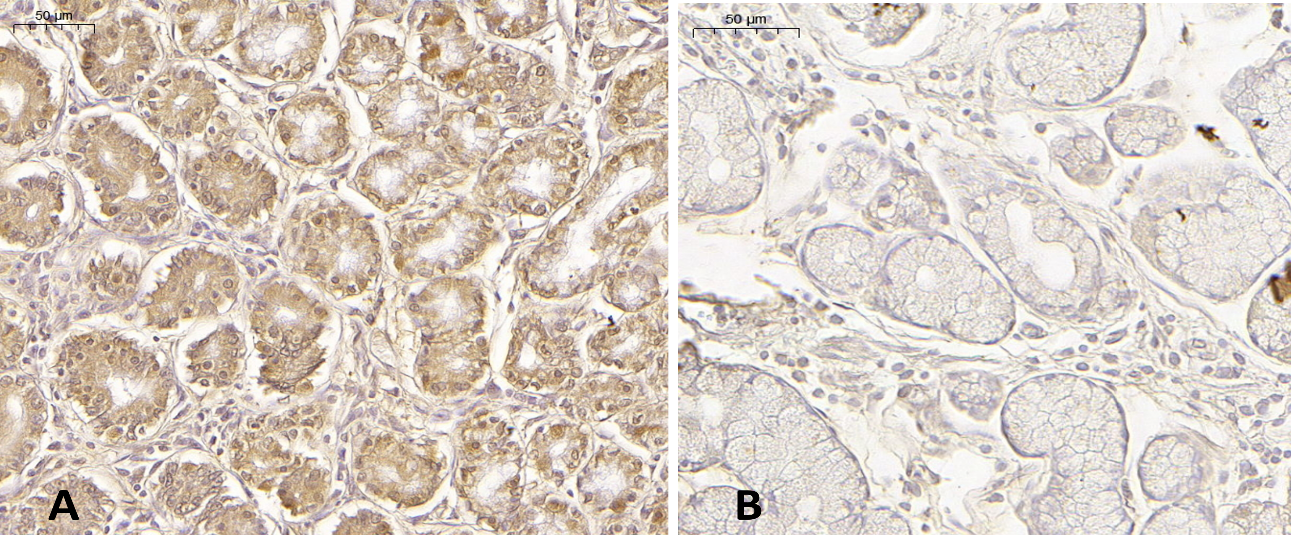


Supplementary Figure 2. GALE immunohistochemistry. A) Positive adjacent normal gastric tissue (A), negative adjacent normal gastric tissue (B). Scale bar: 50 μm.

Supplementary Table 1. Paired comparation of GALE staining in neoplastic cells and gastric non-transformed adjacent tissue.

| Neoplastic  Non-cancerous | UDP glucose 4-epimerase ^(+)^ | UDP glucose 4-epimerase ^(-)^ | *P* Value |
| --- | --- | --- | --- |
| UDP glucose 4-epimerase ^(+)^ | 16 (45.8) | 13 (37.1) |  |
| UDP glucose 4-apimerase ^(-)^ | 5 (14.2) | 1 (2.9) | 0.3662 |

Supplementary Table 2. Validation Cohort. Association between the expression value of UDP glucose 4-epimerase mRNA with the main clinical-pathological characteristics of patients with gastric adenocarcinoma.

| **Clinical data** | **UDP glucose 4-epimerase (+)** | **UDP glucose 4-epimerase (-)** | ***P***  **Value** |
| --- | --- | --- | --- |
|  | **n (%)**  170 (41.3) | **n (%)**  241 (58.3) |  |
| **Age (years) ^a^** |  |  | 0.26 |
| <60 | 122 (30.34) | 162 (40.3) |  |
| ≥60 | 43 (10.7) | 75 (18.65) |  |
| **Sex ^b^** |  |  | 0.14 |
| Female | 52 (12.65) | 91 (22.14) |  |
| Male | 118 (28.71) | 150 (36.5) |  |
| **Surgical Staging ^c^ (TNM)**  (I and II)  (III end IV) | 87 (22.53)  74 (19.17) | 90 (23.31)  135 (34.97) | 0.007 |
| **Lymph node Involvement ^d^**  YES  NO | 103 (28.37)  46 (12.67) | 155 (42.7)  59 (16.25) | 0.556 |
| **Nodal Status ^e^**  >0,3  <0,3 | 52 (14.44)  94 (26.11) | 97 (26.94)  117 (32.5) | 0.081 |
|  |  |  |  |
| **Histological**  **Grade ^f^**  GI + GII  GIII+GIV | 81 (20.6)  83 (21.1) | 77 (19.6)  152 (38.6) | <0.001 |
| **Lauren Classification ^g^**  INTESTINAL  DIFFUSE | 71 (30.2)  25 (10.6) | 101 (43)  38 (16.2) | 0,8815 |
| **Radiotherapy ^h^**  YES  NO | 21 (10.34)  70 (34.48) | 28 (13.8)  84 (41.37) | 0.86 |
| **Relapse ^i^**  YES  NO | 22 (10.18)  75 (34.72) | 22 (10.18)  97 (44.9) | 0.498 |
| **H. Pylori Infection ^j^**  YES  NO | 10 (5.74)  72 (41.37) | 9 (5.17)  83 (47.7) | 0.634 |

Fisher’s exact test. A) Age was analysed in 402 cases. B) Age was analysed in 411 cases. C) Surgical staging was analysed in 386 cases. D) Lymph node involvement was analysed in 363 cases. E) Nodal status was analysed in 306 cases. F) Histological grade was analysed in 393 cases. G) Lauren Classification was analysed in 235 cases. H) Radiotherapy was analysed in 203 cases. I) Relapse was analysed in 216 cases. J) H. Pylori infectionwas analysed in 174 cases.

**A B**

**C** **D**
 ****

Supplementary Graph 1. Associations with the outcome parameters. Overall survival (p = 0.665) (A) and disease-free survival (p = 0.228) (B) of the shunt cohort relative to the overall survival validation cohort (p = 0.605) (C) and disease-free survival (p = 0.637) (D).
